# Supplementary material for: Superior polarization retention through engineered domain wall pinning
Source: Nat Commun. 2020 Jan 17;11:349. doi: 10.1038/s41467-019-14250-7 (PMC6969134; doi:10.1038/s41467-019-14250-7)
Supplement: Supplementary file 1 — Supplementary Information [file 41467_2019_14250_MOESM1_ESM.pdf]

Supplementary Information

Superior polarization retention through engineered domain wall pinning

Zhang et al.

Supplementary Note 1

The annular bright field STEM image in Fig. S1 shows the distribution of defective nanoregions in the BFO thin films. As can be seen clearly, the defective nanoregions pervade the entire film thickness with a homogeneous distribution. Statistics can be extracted from Fig. S1 to calculate the density of defective nanoregions. There are 48 defective nanoregions within a film area of 1659 nm^2 (46.22×35.9 nm^2). Therefore, the density for the defective nanoregion is 48÷1659 nm^2=0.0289 nm^{-2}. If we draw a line along the *z* direction, the average number for the defective nanoregions this line traverses is around 6, so 6÷35.9 nm=0.167 nm^{-1} can be assumed as the line density for the defective nanoregions along the *z*-axis. For a domain with a diameter of 20 nm, which is the smallest visible size in this experiment, the number of the defective nanoregions that are pinning the domain wall can be estimated as follows. One assumption is made here: the density of defective nanoregion in the *x*-*z* plane is the same as of that in the *x*-*y* plane. According to the equation of a circle ($S=\pi r^2$), in the *x*-*y* plane, the area of a 20 nm circular domain and a defective nanoregion (approximated as a circle) are S_{domain}=314 nm^2 and S_{defect} =78.5 nm^2. Considering the density of defective nanoregions, a 20 nm circular domain area in the *x*-*y* plane can cover 9 defective nanoregions (314 nm^2×0.0289/nm^2≈9) assuming all defective nanoregions are zero-dimensional without area. If the area S_{defect} is taken into consideration, only 4 defective nanoregions can fit into the 20 nm domain *x*-*y* plane (S_{domain}÷$S_{defect=}$4), which means at least 5 defective nanoregions are located around the domain wall pinning it (9-4=5) in the *x*-*y* plane. If we multiply the line density of defective nanoregions along the *z*-axis (0.167/nm) by the thickness of the film (60 nm), a 60-nm long line can traverse 10 defective nanoregions along the *z*-axis (0.167 nm^{-1}×60 nm≈10). Given that the domain wall is a three-dimensional object, if we multiply the two numbers for defective nanoregions the 20-nm domain wall can traverse in *x*-*y* plane and *z*-axis, the total number of defective nanoregions that are pinning the domain wall in a three-dimensional space can be obtained, which is 50 (5×10=50). Therefore, even for the smallest domain created in the experiment, the density of these defective nanoregions is high enough to provide effective pinning to the adjacent domain wall. For a larger domain with a diameter of 90 nm, around 100 defective nanoregions are pinning the domain wall, which is also high enough to provide enough pinning to the domain wall.

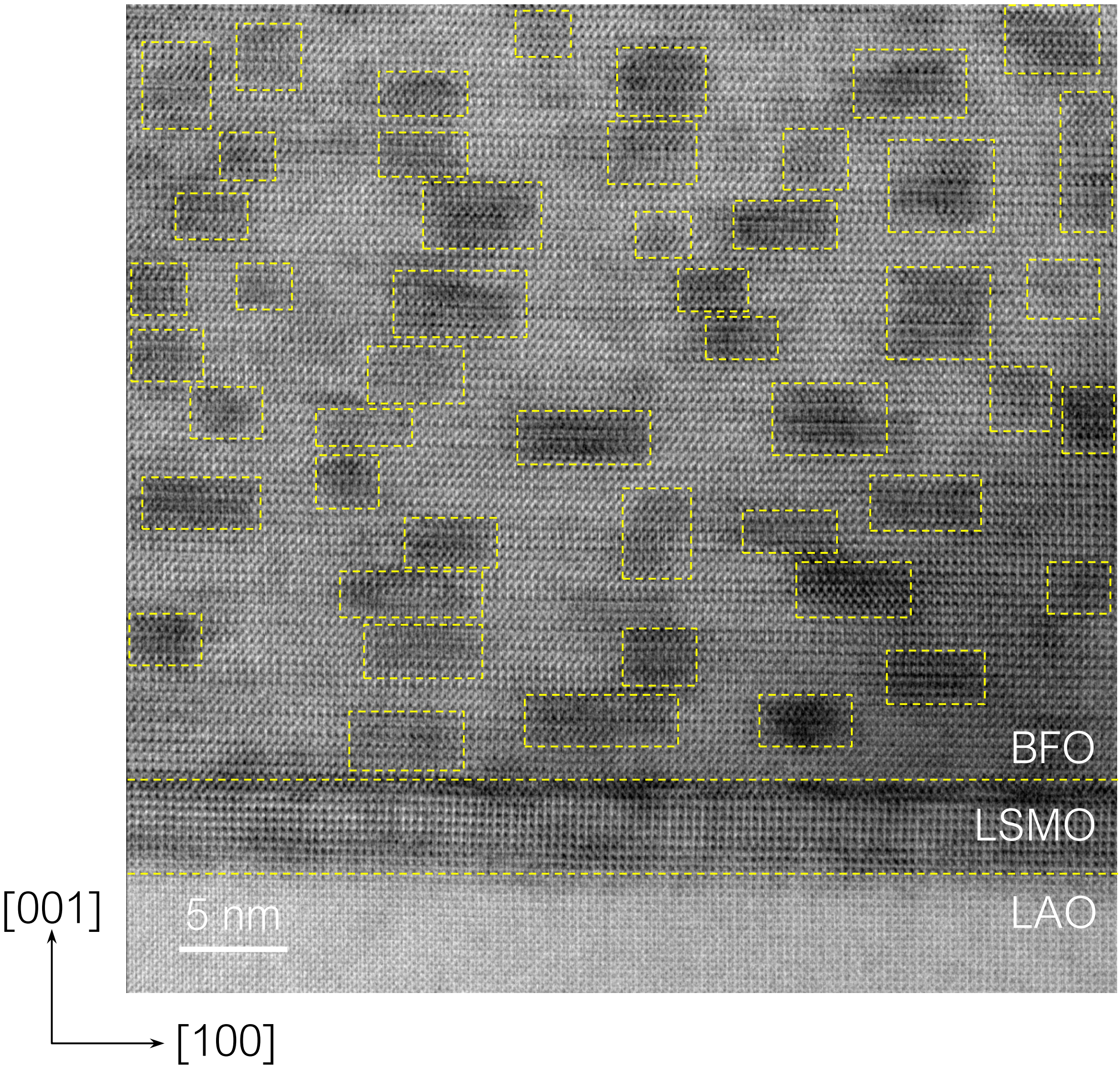

Supplementary Figure 1. Annular bright field scanning transmission electron microscopy (STEM) image of the sample. Defective nanoregions are denoted in yellow boxes.

Supplementary Note 2

Precise measurement of the domain size is of great importance for the present study, so in the following, we outline our approach and compare it with other approaches. Many conventional methods are not accurate enough to obtain very precise domain diameters. Typically, a line-profile that crosses the circle centre is manually selected and then two points on this line-profile with minimum piezoresponse are determined. Therefore, the domain diameter is determined by the distance between these two points. This approach has several disadvantages. The first is that the domain centre is approximately estimated. If there is a shift to the real centre, the diameter might be a chord that is shorter than the diameter. The second is that sometimes the shape of the domain is not a perfect circle, so an average of the vertical and horizontal diameters is not precise. Moreover, an average value of a horizontal and a vertical diameter is not statistically accurate to represent the real average diameter of a written domain. Finally, this method is significantly affected by tip conditions. When the tip is blunt, the image of the domain wall becomes thicker, and from the domain wall centre to the left/right side, the piezoresponse progressively increases. Thus, it is not easy to pinpoint the centre of the domain wall by looking for the lowest piezoresponse as the concave of the line profile becomes flattened. Consequently, a more objective approach which can solve the above problems is required.

Fig. S2 shows theoretically how tip conditions can affect the accuracy of determining the centres of domain walls. Fig. S2 (a)-(b) schematically show the domain wall imaging process with a sharp tip and a blunt tip, respectively. When a sharp tip passes a domain wall, the shape or contour can be nicely imaged with an apparent minimum in the PFM amplitude, as shown in Fig. S2 (a). In comparison, when a blunt tip passes the domain wall, the apparent decrease in amplitude is smaller than the real value and the minimum value is difficult to pinpoint especially when the minima are very close. Fig. S2 (c)-(d) show the way to determine the diameter, which is to measure the distance between the two minima. It should be noted that a blunt tip can lead to a larger full width at half maximum (FWHM), which means a larger error bar. Fig. S2 (e)-(f) are the real line-profile data by a sharp and a blunt tip, respectively. As can be seen in Fig. S2 (e), two minima can be easily determined in the line profile imaged by a sharp tip. By contrast, in Fig. S2 (f), because of the increased contact area between a worn tip and the sample as well as a limited scanning resolution (256 × 256 pixels here), the line profile misses the real minima and shows two flattened valleys instead. As a consequence, it would be advantageous to use a method that can pinpoint the centre of domain walls regardless of the tip conditions.

In Fig. S3, the model for 2D-Gaussian fitting and modified 2D-Gaussian fitting are drawn in (a) and (b) respectively. For the 2D-Gaussian method, only the two valleys corresponding to the two local minima for piezoresponse are fitted using the following equation:

$$Z = Z_0 + Aexp^{\left[-\frac{\left[\sqrt{(x-x_C)^2+(y-y_C)^2}-w_1\right]^2}{2w_2^2}\right]} \quad (1)$$

where Z_0 and A are constants, x_c and y_c are coordinates for the circle centre, w_1 is the expected value for this Gaussian distribution, w_2 is the standard deviation.

This 2D-Gaussian method works perfectly if the tip is sharp. While, for an image imaged by a blunt tip, the 2D-Gaussian method cannot precisely pinpoint the positions for domain wall centres. Then we use a modified 2D Gaussian fitting that also fits a very broad valley caused by the piezoresponse difference inside and outside the domain wall, in which not only the piezoresponse difference can be normalized but also the positions for domain wall centres be pinpointed. The equation is shown below:

$$Z = Z_0 + Aexp^{\left[-\frac{\left[\sqrt{(x-x_C)^2+(y-y_C)^2}-w_1\right]^2}{2w_2^2}\right]} + Bexp^{\left[-\frac{\left[(x-x_C)^2+(y-y_C)^2\right]^2}{2w_3^2}\right]} \quad (2)$$

where B is a constant and w_3 is the standard deviation.

Then the diameter of the domain can be calculated as:

$$D = 2w_1S_f \quad (3)$$

$$S_f = \frac{A_{image}}{R_{pix}} \quad (4)$$

where D is the diameter and S_f is the scaling factor which can be obtained from the ratio between the image area A_{image} and the pixel resolution of the image R_{pix}.

Also, the diameter of the effective tip-sample contact area can be calculated by the following equations:

$$D_{eff} = S_f 2\sqrt{2ln2}w_2 \quad (5)$$

where D_{eff} is the diameter for effective tip-sample contact area or can be regarded as the tip resolution.

A detailed comparison for the fitting results using a 2D Gaussian and a modified 2D-Gaussian method is shown in Fig. S4. Fig. S4 (a) and (b) are horizontal line-profiles of the white line (in the OOP PFM amplitude image in the inset), imaged by a sharp tip and a blunt tip, respectively. Fig. S4 (c)(e) and (d)(f) are the corresponding fitting results for (a) and (b) using two different methods. In Fig. S4 (c)-(f), red, purple and blue curves fit the individual local valleys while the green curve is a cumulative fitting. By combining a multitude of line-profiles going through the circular domain centre, 2D fitting contour mappings and corresponding 2D residual mappings for the raw data are then obtained (Fig. S4 (g)-(n)). Fig. S4 (c) clearly shows that the 2D Gaussian method works perfectly for the line profile imaged by a sharp tip as the two local minima are pinpointed (denoted by the blue dash lines) and the contour of two valleys are also fitted nicely. The diameter is 82 nm. By contrast, in

Fig. S4 (d), for a blunt-tip-imaged line profile, the 2D Gaussian method cannot determine the local minima precisely and tends to narrow the distance between these two minima, which is the diameter, as marked by the pink dash lines. The diameter is 72 nm, 10 nm smaller than 82 nm. From the 2D fitting results and their residuals, it is also clear that the 2D Gaussian is not suitable for the result imaged by a blunt tip. The residual, which is the difference between the raw data and the fitting result, is a good indication for the reliability of the fitting result. In Fig. S4 (l), the amplitude is very high showing a yellowish colour for the contour around the domain wall, which means the fitting is not good. As a comparison, with a modified 2D Gaussian method, regardless of the tip condition, the domain centres can always be pinpointed as shown in Fig. S4 (e)-(f), and the diameter values are the same (82 nm). The 2D fitting mapping and residuals indicate the same. The D and D_{eff} can also be calculated following Eq. (3)-(5). The image size is 2.1 μm by 2.1 μm with a 256 × 256 pixels resolution, so the S_f is 8.203125 nm/pixel. The w_1 for Fig. S4 (g)-(j) are 5.13, 4.35, 5.15 and 5.17, respectively. The w_2 for Fig. S4 (g)-(j) are 0.96, 2.01, 0.93 and 1.35, respectively. So with a 2D Gaussian fitting, a sharp tip (D_{eff1}=18.5 nm) leads to a fitted diameter of 84.2 nm while a blunt tip (D_{eff2}=38.8 nm) leads to a fitted diameter of 71.3 nm. Therefore, the 2D Gaussian method cannot generate an accurate value for domains imaged by a blunt tip. In comparison, whether using a sharp or a blunt tip, the modified 2D Gaussian method can reliably pinpoint the centre of domain walls and generate relatively accurate values. The obtained diameters are almost the same, i.e. 84.5 nm by a sharp tip and 84.9 nm by a blunt tip. Furthermore, 379 domains of various sizes imaged by 19 sharp tips were analyzed using this modified 2D Gaussian method, and the diameter of the effective tip-sample contact area or tip resolution was 18.1 nm. It is known that the domain wall width is on the unit-cell level (one unit cell is ~ 4 Å)[1, 2], the domain wall width imaged by our tips (~18 nm) is limited by the tip radius. In this case, considering the tip radius (< 30 nm), domain diameters smaller than 30 nm are not reliably accurate and very likely to be smaller than the measured values.

In Fig. 4 of the main manuscript, the smallest and the second smallest domain diameters are fitted with a fixed w_2 value. Because of the small sizes, the domains of these two categories sometimes shrink to a dot, which makes defining clear domain wall boundaries demanding. It is assumed that during one scanning, the tip resolution does not change, which means a constant w_2 value. Each array of domains (e.g. the t=0 h array) is cropped out of a large 2.1 μm × 2.1 μm area containing 60 fabricated domains (10 domains for each domain size). At each critical time spot, more than 30 domains were fitted separately in one image to get an average w_2 value. Then the 2D Gaussian method is used to fit these smaller domains with a fixed w_2 value.

Fig. S5 shows the fitting results for the bluntest tip used in all measurements from t=0 h to t=8904 h in Fig. 4a. Fitted by 2D Gaussian method, the D_{eff} is 27 nm. As a comparison, fitted by the modified 2D Gaussian method, the D_{eff} is 21.6 nm which is not too much larger than the average value for D_{eff} that is around 18 nm (as discussed in Fig. S4). So, the error bars for each data set shown in Fig. 4a should be at the same level.

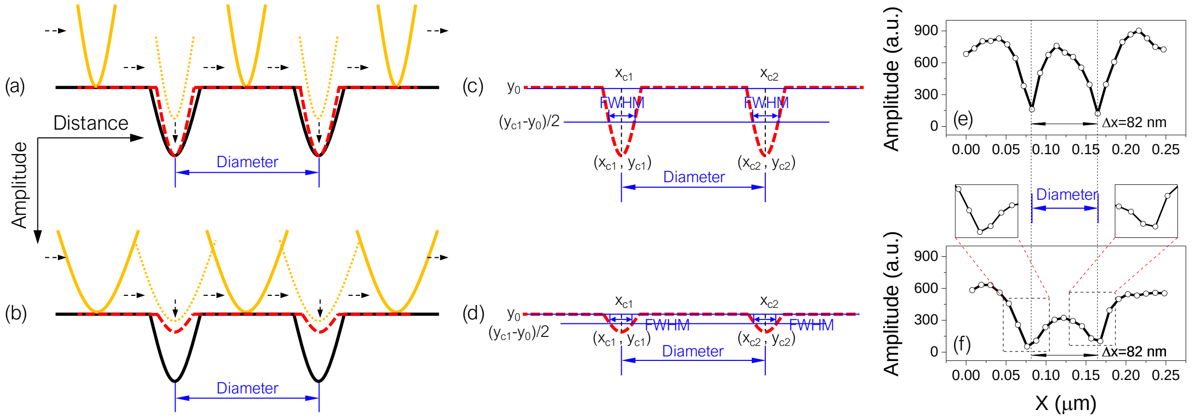

Supplementary Figure 2. The tip condition effect on the accuracy of determining centres of the domain walls. (a)-(b) schematics for imaging the domain walls using a sharp tip and a blunt tip. (c)-(d) ideal contour imaged by a sharp tip and a blunt tip. (e)-(f) The real line-profile data of circular domain walls by a sharp tip and a blunt tip. In the insets of (f), the flattened valleys in the line profile imaged by a blunt tip are shown.

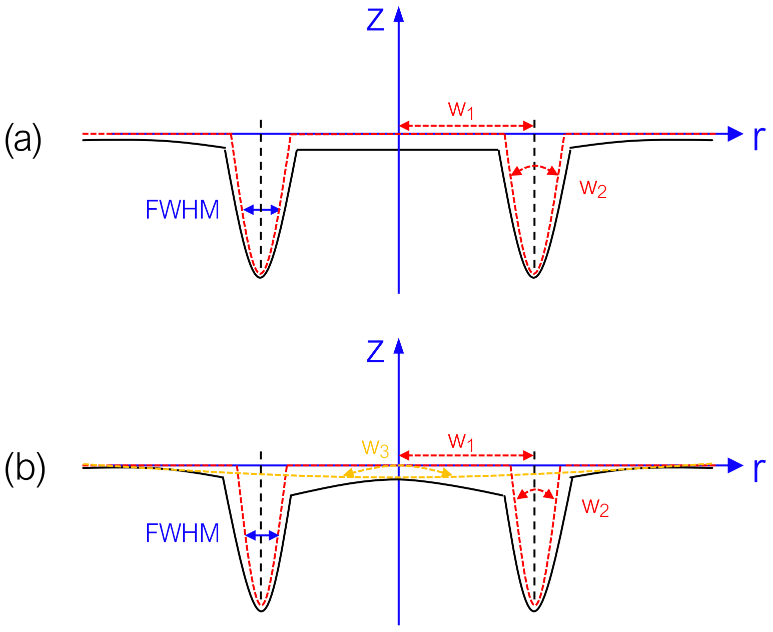

Supplementary Figure 3. A schematic to show the comparison of the 2D Gaussian method and the modified 2D Gaussian method. (a) The schematic for the 2D Gaussian method. (b) The schematic for the modified 2D Gaussian method.

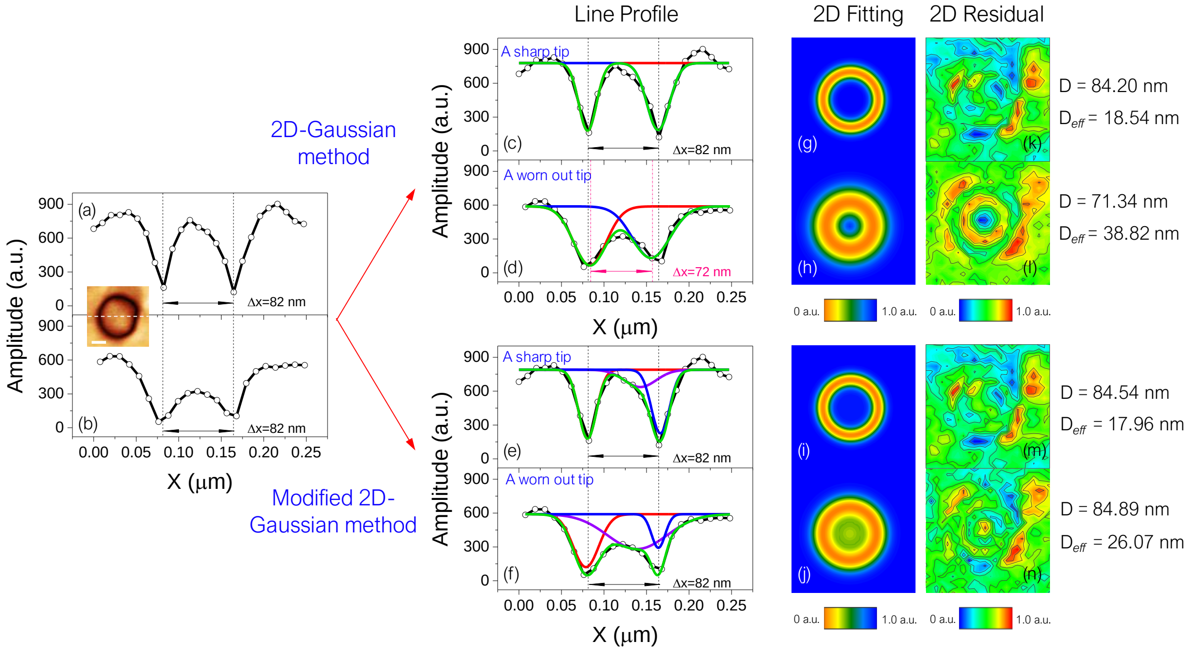

Supplementary Figure 4. Fitting results comparison between 2D-Gaussian method and modified 2D-Gaussian method. (a)-(b) horizontal line-profiles for lines that cross the domain wall in an OOP PFM amplitude image using a sharp tip and a blunt tip respectively. The scale bar is 30 nm. (c)-(d) fitting results for a sharp-tip-imaged line-profile and a blunt-tip-imaged line-profile by the 2D-Gaussian method. (e)-(f) fitting results for a sharp-tip-imaged line-profile and a blunt-tip-imaged line-profile by the modified 2D Gaussian method. (g)-(j) corresponding 2D fitting mappings for the domain. (k)-(n) corresponding 2D residual mappings for the domain.

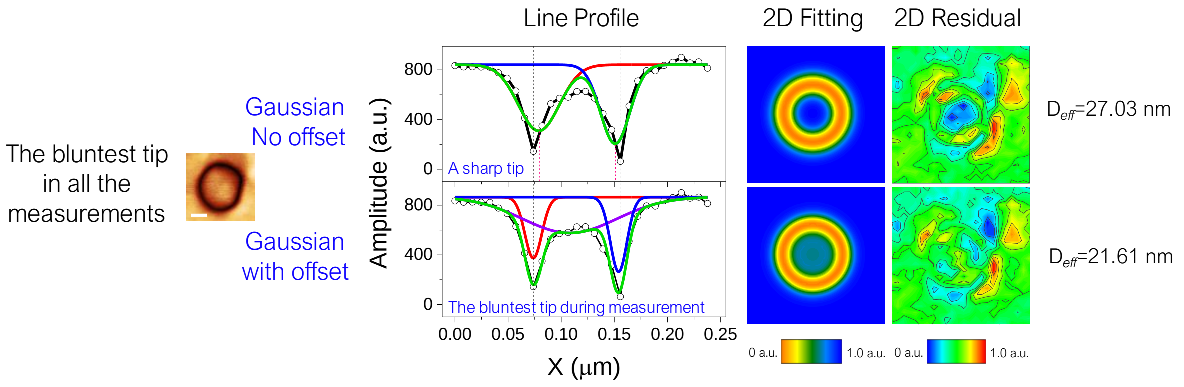

Supplementary Figure 5. Analysis of the bluntest tip used in all the measurements in Figure 3. Line profile results, 2D fitting mappings and 2D residual mappings fitted by the 2D-Gaussian method and the modified 2D Gaussian method. The scale bar is 30 nm.

Supplementary Note 3

Fig. S6 shows the tip-voltage dependence and pulse-time dependence of domain sizes. In Fig. S6a the domain diameters as a function of tip voltage are shown in an OOP amplitude PFM image. The tip voltages applied were -10 V, -9 V, -8 V, and -7 V from the bottom array to the top array at a fixed pulse duration of 300 ms. Lower tip voltages from -6 V to -1 V were also applied in this image, but these voltages were not high enough to form stable domains for detection. Fig. S6b and Fig. S6c show all the data points for Fig. 3a (the relationship between the domain diameter and the pulse time) and Fig. 3b (the relationship between the domain diameter and the tip voltage), respectively.

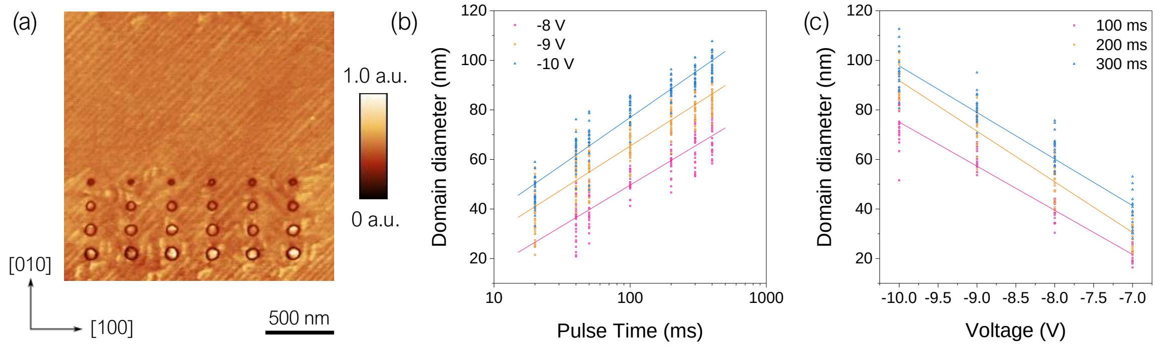

Supplementary Figure 6. Domain diameter as a function of pulse time and tip voltage. (a) The out-of-plane amplitude PFM image of domains fabricated with a fixed pulse duration of 300 ms at different tip voltages (-10 V, -9 V, -8 V and -7 V, respectively from the bottom line to the top line). Lower voltages from -6 V to -1 V with an increment of 1 V were also applied but no domains were imaged. (b) Domain diameters in relation to pulse time durations (all the data points for Figure 3a). (c) Domain diameters in relation to tip voltages (all the data points for Figure 3b). All error bars presented in this figure represent the standard deviations of the average values of 3~4 data sets at each fixed voltage and pulse duration.

Supplementary Note 4

The experiment in Fig. 4 was repeated under the same conditions in a different region of the sample, as shown in Fig. S7a. The OOP PFM amplitude images recorded at different times (at 0 h, 6887 h, 7198 h, 7531 h, 7863 h, 8056 h, 8243 h, 8561h and 8900 h) show that the diameters for domains of various sizes are almost constant. Then the diameters for each domain in one array are obtained by using the modified 2D Gaussian fitting method and then are shown in Fig. S7 (b). The calculation details have been explained in Fig. S4. Also, the diameters for a domain fabricated by -9 V at 300 ms in the first 2000 hours are also shown. For larger domains (switched by 300 ms, 200 ms, 100 ms, 50 ms and 20 ms), there are some fluctuations (mainly caused by different tip conditions and imaging qualities) but not too large compared to diameter values, but the domain diameters almost remain around the same values over time. For instance, for the domain switched with a pulse time of 200 ms, the diameter is 79.5 nm at t=0 h and becomes 79.0 nm at t=8900 h. For smaller domains (switched by 5 ms, 10 ms), the diameters recorded at 0 hour are larger because the tip used at 0 hour was not very sharp, which results in a blurry image and a wider domain wall width than expected. For the later time intervals (from t=6887 h to t=8900 h), the diameters remain at the same level. In summary, over time, the polarization for domains with various sizes remains almost unchanged and stable. Also, this robust retention is independent of the initial domain diameters.

The temperature stability of the written domains is also of importance for practical electronic information storage applications. Therefore, we have performed polarization retention experiments at various temperatures as follows. First, nanoscale domains were written by SPM tips at room temperature. Next, the sample was heated to a given target temperature and held at this temperature for 30 min. The sample was then cooled down to room temperature and polarization retention property was checked using the out-of-plane PFM signal. As can be seen from Fig. S8 (a), the polarization retention can be maintained from room temperature (25 ^{o}C) up to 175 ^{o}C with very clear domain structures. Temperatures higher than 175 ^{o}C may cause chemical changes to the sample so in this experiment we fixed the upper temperature to be 175 ^{o}C. It should be noted that the diameters slightly decrease with the increase of the temperature, as shown in Fig. S8 (b). Here the average diameters of the six domains are the data points and their standard deviations are the error bars. The average decrease of the diameters/polarization at 175 ^{o}C is around 10 percent compared to that of the domains at 25 ^{o}C. This shows that the polarization retention is quite stable over a wide temperature range.

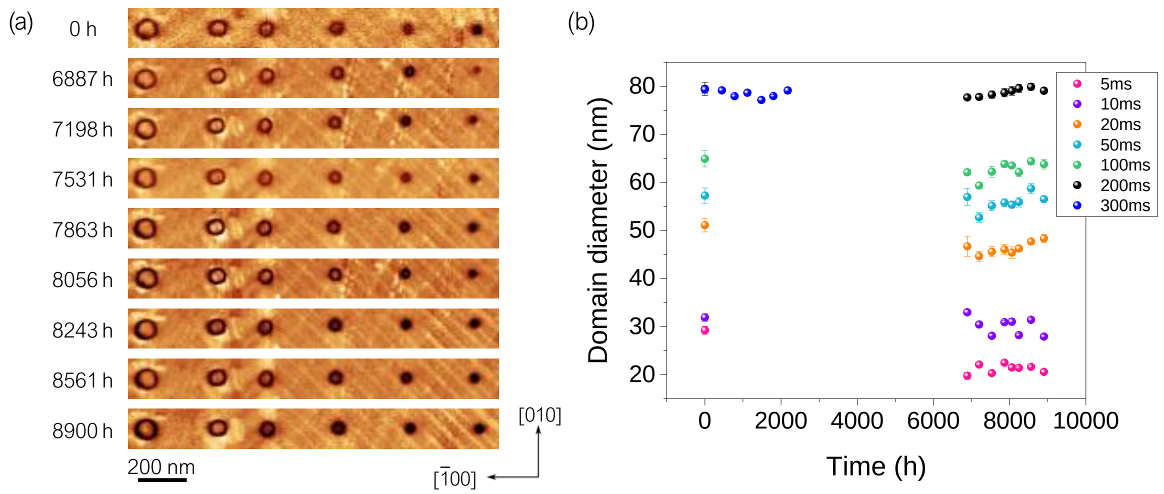

Supplementary Figure 7. Polarization retention behaviour over time for domains poled in a different region compared to Fig 3. (a) The Out-of-plane PFM amplitude images of domains with different diameters recorded at 0 h, 6887 h, 7198 h, 7531 h, 7863 h, 8056 h, 8243 h, 8561h and 8900 h respectively. The domains were fabricated by a tip voltage of -9V with different pulse duration times (5 ms, 10 ms, 20 ms, 50 ms, 100 ms and 200 ms). (b) Diameters for domains of various sizes as a function of time. All the data are extracted from (a) and fitted by the modified 2D-Gaussian method. The domains fabricated with a pulse time of 300 ms at -9 V with a different tip exhibit the no-decay polarization retention behaviour during the preliminary 2000 hours. The error bar shown here represents the standard error for each data point fitted by the modified 2D-Gaussian method.

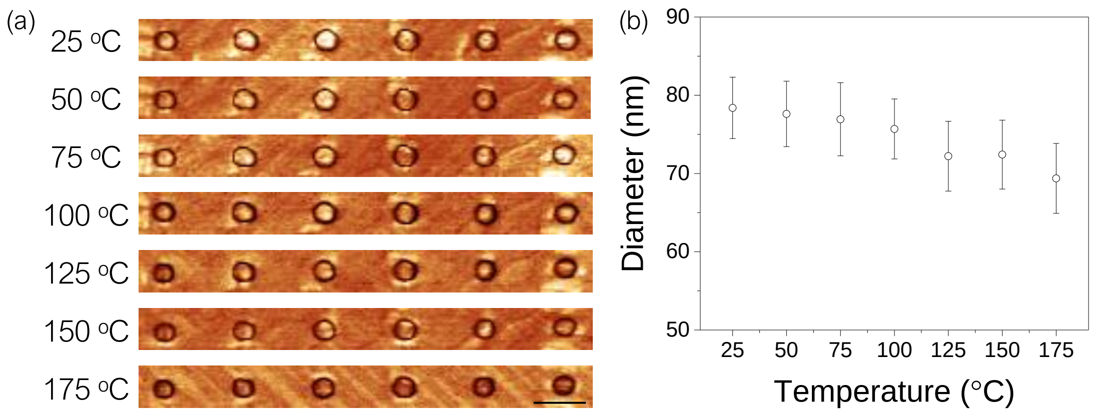

Supplementary Figure 8. Polarization retention at different temperatures. a Polarization retention behaviour after being heated at various temperatures. **b** The average diameter as a function of temperature. The scale bar is 200 nm. The error bar shown here represents the standard deviation.

Supplementary Note 5

To study the retention enhancement obtained from the *R/T* mixed phase boundaries as reported[3], a comparison experiment was done by fabricating domains on *R'/T'* mixed phase boundaries and *T'* matrix in our BFO system, which is shown in Fig. S9. A tip voltage of -5.5 V was used to create the *R'* stripes, as can be seen both in morphology and OOP PFM amplitude images, and then two arrays of domains were fabricated on *R'/T'* mixed phase boundaries and *T'* matrix using a tip voltage of -9V at 20 ms, respectively.

The OOP PFM amplitude images recording the domain polarization retention behaviour for domains on *R'/T'* phase boundaries and *T'* matrix are shown in Fig. S10 (a). As can be seen, there is not too much difference for the retention in two types of domains from t=0 h to t=8921 h. We calculated diameters for 5 domains on *R'/T'* matrix and 5 domains on *T'* matrix, and the normalized retained polarization values for them are shown in Fig. S10 (b). From Fig. S10 (b), It can be seen that there is little difference for these two types of domains (98% retained for domains on *T'* matrix and 94% retained for domains of *R'/T'* mixed phase). The domains on *T'* matrix seem to have a slightly better retention property. It is understandable as electrically induced *R'* domains are not energetically stable compared to as-grown *R'* domains. Over time, there is some back-switching for electrically fabricated *R'* domains, which leads to a release of local strain and therefore causes a slight change in the domain diameters. Our result therefore does not contradict the literature[3] as the electrically induced *R'* and as-grown *R* domains have different energy status.

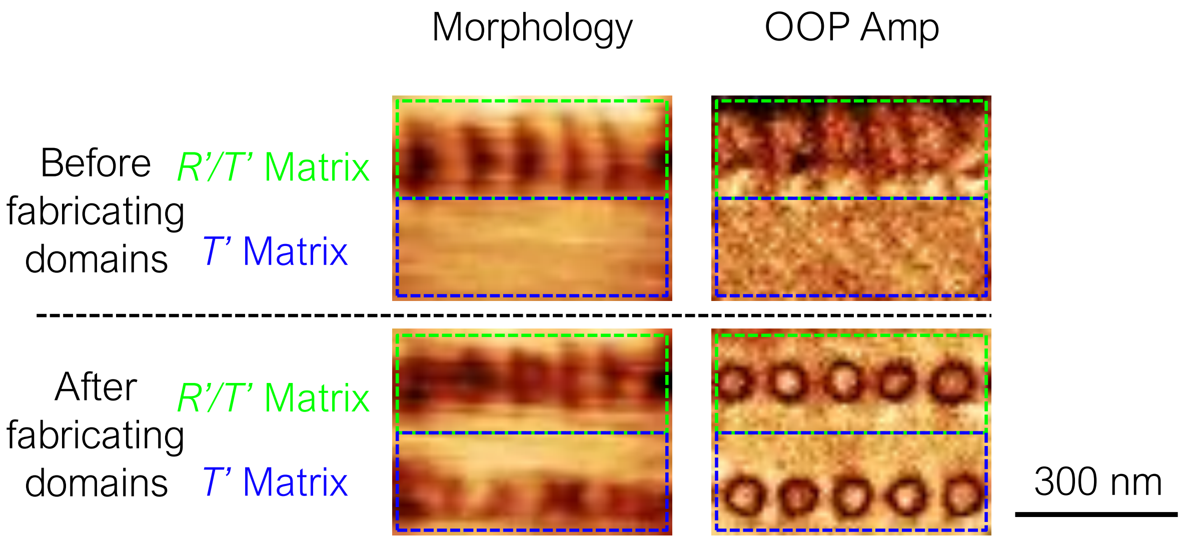

Supplementary Figure 9. Topography and OOP PFM amplitude images to show domains grown on *R'/T'* mixed phase boundaries and *T'* matrix.

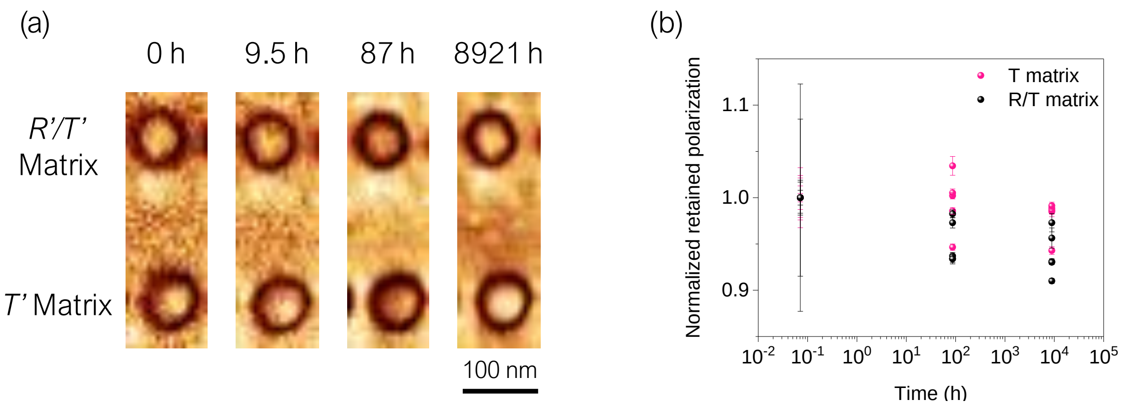

Supplementary Figure 10. Polarization retention comparison for domains fabricated on *R'/T'* phase boundaries and *T'* matrix. (a) OOP PFM amplitude images for domains fabricated on *R'/T'* matrix and *T'* matrix at 0 h, 9.5 h, 87 h and 8921 h. (b) Normalized retained polarization as a function of time.

Supplementary References

1. Jia, C.-L., Mi, S.-B., Urban, K., Vrejoiu, I., Alexe, M., Hesse, D. Atomic-scale study of electric dipoles near charged and uncharged domain walls in ferroelectric films. *Nat. Mater.* **7**, 57-61 (2008).
2. Catalan, G., Seidel, J., Ramesh, R., Scott, J. F. Domain wall nanoelectronics. *Rev. Mod. Phys.* **84**, 119 (2012).
3. Huang, Y. C., *et al.* Giant enhancement of ferroelectric retention in $BiFeO_3$ mixed-phase boundary. *Adv. Mater.* **26**, 6335-6340 (2014).
